# Status of lipid and lipoprotein in female breast cancer patients at initial diagnosis and during chemotherapy

**DOI:** 10.1186/s12944-018-0745-1

**Published:** 2018-04-20

**Authors:** Xin Li, Zi-li Liu, Yu-tuan Wu, He Wu, Wei Dai, Bilal Arshad, Zhou Xu, Hao Li, Kai-nan Wu, Ling-quan Kong

**Affiliations:** Department of Endocrine and Breast Surgery, The First Affiliated Hospital of Chongqing Medical University, Chongqing, 400016 China

**Keywords:** Breast cancer, Dyslipidemia, Adjuvant chemotherapy

## Abstract

**Background:**

The lipid profile status among breast cancer patients at initial diagnosis and during chemotherapy remain controversial. The aim of this study is to study the status of lipid and lipoprotein in female breast cancer patients at initial diagnosis and during chemotherapy.

**Methods:**

We conducted a retrospective cohort study of the status of the lipid and lipoprotein in 1054 primarily diagnosed breast cancer patients and 2483 normal controls with age stratification, from July 2015 to October 2016. At the same time, the status of lipid and lipoprotein were also analyzed among 394 breast cancer patients before and after adjuvant chemotherapy.

**Results:**

The incidence of dyslipidemia was significantly lower in breast cancer group(42.98%) compared to normal group(58.28%)(*P* < 0.001). The levels of total cholesterol (TC), triglycerides (TG), HDL cholesterol (HDL-C), LDL cholesterol (LDL-C) among breast cancer group were significantly lower compared to normal control group (*P* < 0.05). With age stratification, the levels of TC and LDL-C in breast cancer group were still significantly lower than those in control group (*P* < 0.001). And the levels of TC, TG, LDL-C, apolipoprotein B were significantly higher among post chemotherapeutic patients compared to prechemotherapeutic patients, however HDL-C and Apo-A1 levels were contrary.

**Conclusions:**

Breast cancer patients have lower incidence of dyslipidemia compared to normal populations. However, the situation of dyslipidemia may become worsened after chemotherapy. Therefore, lipid monitoring and dyslipidemia prevention and treatment should be conducted for breast cancer patients at initial diagnosis and during chemotherapy.

## Background

Breast cancer is the most general diagnosed cancer and the second leading cause of cancer-related death among women worldwide [1]. The female breast cancer mortality is down to 36% from peak rates due to early diagnosis and treatment [2]. However, it is still crucial to investigate the relative hazardous factors and methods to improve prognosis.

An unbalanced lipid profile with high total cholesterol (TC), low-density lipoprotein-cholesterol (LDL-C), triglycerides (TG), and low high-density lipoprotein-cholesterol (HDL-C), apolipoprotein A1(Apo-A1), apolipoprotein B (Apo-B) is an established risk factor of cardiovascular diseases [3, 4]. LDL-C has been successfully treated by lipid-lowering therapies. Apolipoproteins are crucial for the development of HDL and LDL lipoprotein complex. Moreover, Apo-B is considered as a better indicator of cardiovascular disease (CVD) compared to LDL-C [5]. Apo-A1 binding protein accelerates cholesterol efflux from endothelial cells and regulates angiogenesis [6]. Plasma lipids and lipoproteins are influenced by environmental factors, including weight and diet, and are closely associated with breast cancer risk factors which suggest the role of lipids in causing breast cancer. A study suggested that higher mammographic density considered to be a strong risk factor of breast cancer [7], which is associated with increased HDL-C levels and decreased LDL-C levels [8]. Furthermore, HDL-C level is associated with several other breast cancer risk factors [9]. Several studies have reported the association between lipids and breast cancer. However, the results are controversial. Some prospective clinical studies reported that high levels of TC and HDL-C may increase breast cancer incidence [9–11]. However, others have suggested that low TC and HDL-C levels could increase breast cancer risk [12, 13]. Few researches have studied the status of lipid in breast cancer patients before and after chemotherapy. Derya H B et al. reported that adjuvant chemotherapy may contribute to an increased risk for metabolic syndrome in breast cancer patients and these changes are more profound in pre-menopausal patients [14]. The aim of this study is to investigate the status of serum lipids and lipoproteins in breast cancer patients and normal controls and their changes during chemotherapy.

## Methods

### Subjects

The clinical data of blood lipid status of 1054 primarily diagnosed breast cancer patients and 2483 normal women (as controls) were collected from the electronic medical records of Breast Cancer Center and Chongqing Physical Examination Center of the First Affiliated Hospital of Chongqing Medical University, from July 2015 to October 2016. The data were comparatively analyzed with age stratification. Also the blood lipid status of 394 breast cancer patients were comparatively analyzed before and after chemotherapy. Key exclusion criteria included the patients with history of other malignancy and tumor recurrence and with no pathological reports.

### Evaluate parameters

This study compares the status of lipids among breast cancer patients and normal people. The status of lipid and apolipoprotein of 394 breast cancer patients were evaluated before and after chemotherapy. The biochemical parameters related to dyslipidemia TC, TG, HDL, LDL, Apo A-1, Apo-B were categorized using cut-off values as follows: TC with 5.20 mmol/L, TG 1.7mml/L, HDL-C 0.9 mmol/L, LDL-C 3.1 mmol/L. Dyslipidemia was considered when TC > 5.2 mmol/L or TG > 1.7 mmol/L or LDL-C > 3.1 mmol/L or HDL-C < 0.9 mmol/L.

### Statistical analysis

The data were analyzed using the Statistical Pakage for Social Sciences (SPSS) software version 22.0. Mean (x) and standard deviation (SD) were evaluated using student t-test for the comparison between control and patient groups. *P* < 0.05 was considered statistically significant. The incidence of dyslipidemia among both groups was compared using Chi-square test. The mean values of lipids among both groups were compared using independent sample T test. Finally, the status of blood lipids and apolipoproteins in breast cancer patients before and after chemotherapy were compared using paired-sample T test.

## Results

No significant difference was observed in the mean age of breast cancer group (49.93 ± 10.44 years) and control group (50.16 ± 12.07 years). In our study, We observed the prevalence of preobese or obesity(BMI ≥ 25) was 27.61% in breast cancer patients and 22.43% in control population, respectively. Baseline characteristics of breast cancer patients and control population are shown in Table 1.

**Table 1:** Baseline characteristics of the study population

Characteristics	Breast cancer(*n* = 1054)	Control group(*n* = 2483)	*P*-value
Age, years(Mean ± SD)	49.93 ± 10.43	50.16 ± 12.07	0.569
Body mass index (BMI), kg/m2(Mean ± SD)	23.44 ± 3.16	22.86 ± 3.09	< 0.001
Underweight (BMI < 18.5)(n,%)	53(5.03)	124(4.99)	0.004
Normal (18.5 ≤ BMI ≤ 25)(n,%)	710(67.36)	1802(72.57)	
Preobese (25 ≤ BMI ≤ 30)(n,%)	253(24.00)	503(20.26)	
Obesity (BMI ≥ 30)(n,%)	38(3.61)	54(2.17)	
Stage (n,%)		–	–
I	311(29.51)	–	–
II	456(43.26)	–	–
III	214(20.30)	–	–
IV	4(0.38)	–	–
Missing	69(6.54)	–	–

The incidence of dyslipidemia was significantly lower in breast cancer group compared to control group (*P* < 0.001). The levels of TC and LDL-C in breast cancer group were significantly lower than those in control group (*P* < 0.001). With age stratification, the levels of TC and LDL-C in breast cancer group were still significantly lower than those in control group (*P* < 0.001). Significant statistical difference was observed in the TG level of breast cancer group compared to normal group (*P* < 0.05); While with age stratification, decreased TG level in breast cancer group was found only in 40–49 years group (*P* < 0.05). There was a significant statistical difference in HDL-C level among breast cancer group compared to control group (*P* < 0.001), respectively; While with age stratification, the HDL-C level was significantly higher in normal group in 40–59 years group (*P* < 0.05). The comparison of lipid profiles in breast cancer group and control group are shown in Table 2.

**Table 2:** Comparision of the incidence of lipidemia between breast cancer patients and control group

Parameter		Breast cancer (*n* = 1054)					Control group (*n* = 2483)				
		TC (Mean ± SD, mmol/L)	TG (Mean ± SD, mmol/L)	HDL-C (Mean ± SD, mmol/L)	LDL-C (Mean ± SD, mmol/L)	Dyslipidemia (n, %)	TC (Mean ± SD, mmol/L)	TG (Mean ± SD, mmol/L)	HDL-C (Mean ± SD, mmol/L)	LDL-C (Mean ± SD, mmol/L)	Dyslipidemia (n, %)
Total		4.47 ± 0.93**	1.27 ± 1.02*	1.43 ± 0.36**	2.80 ± 0.81**	453(42.98)**	5.01 ± 0.95	1.33 ± 0.86	1.48 ± 0.32	3.19 ± 0.87	1447(58.28)
Age stratification (years)	20–39	4.04 ± 0.83*	0.95 ± 0.54	1.45 ± 0.34	2.43 ± 0.73	33(25.58)	4.27 ± 0.70	0.94 ± 0.44	1.47 ± 0.29	2.52 ± 0.65	78(22.41)
	40–49	4.32 ± 0.85**	1.14 ± 0.67*	1.43 ± 0.33*	2.69 ± 0.76**	150(34.88)**	4.84 ± 0.84	1.25 ± 0.90	1.49 ± 0.32	3.03 ± 0.78	447(49.72)
	50–59	4.67 ± 1.02**	1.45 ± 1.53	1.42 ± 0.40**	2.94 ± 0.84**	149(52.84)**	5.34 ± 0.92	1.44 ± 0.88	1.51 ± 0.35	3.47 ± 0.86	540(74.07)
	≥60	4.78 ± 0.88**	1.47 ± 0.91	1.45 ± 0.39	3.04 ± 0.79**	121(56.81)**	5.34 ± 0.95	1.59 ± 0.84	1.44 ± 0.31	3.51 ± 0.87	382(75.35)

*p < 0.05 **p < 0.001 comparison of lipid levels and dyslipidemia incidence between breast cancer patients and control group; TC: total-cholesterol, TG: triglyceride, HDL-C: high density lipoprotein-cholesterol, LDL-C: low density lipoprotein-cholesterol

The basic situation of patients received chemotherapy were shown in Table 3, and it recorded the weight changes before and after chemotherapy. TAC chemotherapy regimen (docetaxel, doxorubicin, cyclophosphamide), cycled every 21 days for 6 cycles. AC-T chemotherapy regimen (doxorubicin and cyclophosphamide, cycled every 21 days for 4 cycles. Followed by docetaxel, cycled every 21 days for 4 cycles). CEF chemotherapy regimen(5- fluorouracil Epirubicin and cyclophosphamide),cycled every 21 days for 6 cycles.

**Table 3:** The baseline characteristics of the patients received chemotherapy

Characteristics	Value
Age, years, mean ± SD	48.31 ± 8.80
menopausal status at diagnosis	
premenopausal (n, %)	239(60.66)
post menopausal (n, %)	155(39.34)
Weight	
prechemotherapy, mean ± SD(KG)	56.70 ± 7.64
post chemotherapy, mean ± SD(KG)	58.06 ± 7.74
Chemotherapy regimens	
TAC (n, %)	251(63.71)
AC-T (n, %)	134(34.01)
CEF (n, %)	9(2.28)

Comparison of lipid profiles in 394 breast cancer patients before and after chemotherapy is shown in Table 4. Regarding to various lipid parameters, the differences in lipid levels of TC, TG, LDL-C, Apo-B (increase in blood levels), HDL-C and Apo-A1(decrease in blood levels) were statistically significant before 1st chemotherapeutic cycle compared to the last cycle (*P* < 0.001).The levels of TC, TG, LDL-C, Apo-Bin among primarily diagnosed breast cancer patients before chemotherapy were 4.47 ± 0.91,1.31 ± 1.20,2.78 ± 0.80 and 0.85 ± 0.23, however the levels after chemotherapy, increased to 4.80 ± 0.88,1.77 ± 1.21,3.18 ± 0.81 and 0.97 ± 0.24, respectively (*P* < 0.001).The pre-chemotherapeutic levels of HDL-C and Apo-A1 significantly decreased after chemotherapy(*P* < 0.001). The changes in lipid level among premenopausal and postmenopausal women undergoing chemotherapy were similar to the normal patients.

**Table 4:** Comparision of the status of lipidemia between breast cancer patients with pre and post chemotherapy

Parameter	Pre-chemotherapy group(*n* = 394)		Post-chemotherapy group (*n* = 394)	
	Mean ± SD (mmol/L)	Abnormal (n, %)	Mean ± SD (mmol/L)	Abnormal (n, %)
TC	4.47 ± 0.91**	79(20.05)**	4.80 ± 0.88	119(30.20)
TG	1.31 ± 1.20**	72(18.27)**	1.77 ± 1.21	160(40.61)
HDL-C	1.43 ± 0.36**	20(5.08)*	1.28 ± 0.35	42(10.66)
LDL-C	2.78 ± 0.80**	135(34.26)**	3.15 ± 0.81	203(51.52)
Apo-A1	1.48 ± 0.25**	10(2.54)**	1.39 ± 0.24	35(8.88)
Apo-B	0.85 ± 0.23**	11(2.79)	0.97 ± 0.24	20(5.08)
Dyslipidemia	–	201(45.07) **	–	306(68.61)

*p < 0.05 **p < 0.001 comparision of the status of lipidemia between pre and post chemotherapy among breast cancer patients; TC: total-cholesterol, TG: triglyceride, HDL-C: high density lipoprotein-cholesterol, LDL-C: low density lipoprotein-cholesterol, Apo-A1: apolipoprotein-A1, Apo-B: apolipoprotein-B

## Discussion

In this study, we demonstrate that the incidence of dyslipidemia in breast cancer patients (42.98%) was significantly lower than that in control group(58.28%,*P* < 0.001), and the total serum levels of cholesterol, triglycerides, HDL-C and LDL-C are significantly lower in breast cancer patients than those in normal controls in southwest of China. The cholesterol increased significantly during chemotherapy, except for the decrease in HDL-C.

A study results refers to that the thyroxine(T4) level in initially diagnosed breast cancer patients were significantly higher than those in benign breast diseases patients. And during chemotherapy, the T4, free triiodothyronine (FT3), and free thyroxine (FT4), were significantly lower than in initially diagnosed breast cancer patients [15]. It is well known that increased thyroxin level may decrease cholesterol level [16]. Compared with normal population, the breast cancer patients were in higher cholesterol levels, and the increased cholesterol level among chemotherapeutic patients may be partly related with the increased thyroxin level during chemotherapy which had been found by our previous studies [15, 17].

Currently, some studies have investigated the association between lipids and breast cancer. A large prospective studies in Korea implicated that higher cholesterol increased the breast cancer risk [18]. However, the data from other studies generally do not support the association between cholesterol and breast cancer risk [19–21]. A recent meta-analysis about association of lipid profile levels to breast cancer females implicated no significant differences in the levels of total cholesterol, low density lipoprotein cholesterol between cases and controls [22]. These data support an inverse association between cholesterol levels, which has been previously reported [13, 23–25]. Fiorenza AM and his colleague got the similar results to ours, cancer patients had lower mean total cholesterol, LDL-C, and HDL-C than non-cancer subjects. Patients with metastatic disease had lower total cholesterol and LDL-C than patients without metastasis [26]. Knapp et al. observed low LDL-C and HDL-C in patients with advance breast cancer [27]. An earlier observational results clearly indicated that hypocholesterolemia among cancer patients is due to disease progression [28]. Low LDL-C in malignancy might be explained by an increased demand of cholesterol from neoplastic cells, resulting in increased LDL removal through the enhancement of LDL receptor activity [29, 30]. This pattern of lipid abnormalities is very similar to that observed during the acute-phase response in a variety of acute and chronic diseases [31], and might be due to the release of proinflammatory cytokines [32]. It is possible that lipid abnormalities in cancer patients might represent an acute-phase response due to cytokines delivery by inflammatory cells around the tumor or by the tumor cell itself [33]. An experimental study showed that breast cancer cells consume cholesterol for the promotion and proliferation of breast cancer cells [34].

Although Adjuvant chemotherapy improves both disease-free and overall survival of breast cancer patients, accumulating evidence suggest that chemotherapy may cause significant alterations in the metabolic status of cancer survivors [35]. In our study, we have observed some significant metabolic changes during adjuvant chemotherapy of breast cancer patients such as increase in total cholesterol, triglycerides, LDL-C and Apo B, and decrease in HDL-C and Apo A1. Meanwhile, Apo A1 is the crucial component of HDL and both are protective factors of cardiovascular diseases [36]. Monika Sharma et al. investigate the longitudinal effect of chemotherapy on lipids in the same group of patients by monitoring the serum lipid profiles of 12 breast cancer patients throughout their multi-agent chemotherapy treatments have obtained the similar results of lipids changes before and after chemotherapy [37]. Some hypotheses implicate that chemotherapy may directly cause endothelial dysfunction, leading to cytokine alterations, and hence may cause development of lipids [38, 39]. Further evidence show that cancer-associated adipocytes modify the cancer cell phenotype leading to a more aggressive behavior [40]. An experimental study on mice with elevated circulating levels of 27-hydroxycholesterol(27HC), a primary cholesterol metabolite, increased the metastasis of breast cancer cells to lung [34]. Furthermore, a research found that simvastatin, a highly lipophilic statin, was associated with reduced risk of breast cancer recurrence among Danish women [41]. Thus breast cancer patients taking statins could improve the quality of life and prognosis.

## Conclusions

In summary, breast cancer patients have lower incidence of dyslipidemia compared to normal populations. However, the situation of dyslipidemia may become worsened after chemotherapy. Therefore, lipid monitoring and dyslipidemia prevention and treatment should be conducted for breast cancer patients at initial diagnosis and during chemotherapy.

## Abbreviations

Apo A-1: Apolipoprotein A-1
Apo-B: Apolipoprotein B
HDL-C: High density lipoprotein-cholesterol
LDL-C: Low density lipoprotein-cholesterol
TC: Total cholesterol
TG: Triglyceride
